# The Gold-headed Cane

**Published:** 1860-03

**Authors:** 


### Editors' Table.

The Gold-headed Cane. II.—Under a belief that the reminiscences of the gold-headed cane, introduced in our last number, were quite acceptable to our readers, we proceed to draw from the same source, for their further gratifications at the present time. The narrator had, it will be remembered, told of its having been transferred from Radcliffe to Mead; from a physician more remarkable for good sense and sagacity than for literary attainments to one who was a scholar and a man of refined tastes. Dr. Mead was universally allowed to be a man of great erudition, to be *artis medicæ decus, vitæ revera nobilis*, and who went beyond the nobles of the land in his love for, and patronage of, the "fine arts, polite learning, and a knowledge of antiquity." The gold-headed cane, though it had changed masters, had the satisfaction of returning to the old Home, for Mead not only succeeded Radcliffe in the greater part of his business, but removed to the residence which he had formerly occupied in Bloomsbury Square, at that time the fashionable part of London.

About six months after the death of Radcliffe, the cane was present at a consultation between Sir Hans Sloane, Dr. Cheyne, and Mead, in the case of the celebrated Bishop Burnet, author of the "History of his own Time," and the "Sacred Theory of the Earth." Up to the date of his attack, (of pleurisy,) which proved fatal, the bishop, then seventy-two years of age, had enjoyed good health. As a contribution to personal hygiene, we shall repeat what the patient said to Dr. Mead on this occasion: "In summer I have been in the habit of rising at five in the morning, in the winter at six; and I have always officiated, myself, at prayer, though my chaplains may have been present. I then took my tea in company with my children, and read the Scriptures with them. I have generally spent six or eight hours in my study. The rest of the day has been passed by me in taking exercise, making friendly visits or cheerful meals. But now, to use an expression of my late gracious master, King William, whom I knew well for sixteen years, I feel '*que je tire vers ma fin*,'"—(that I draw near my end.) The presage was soon fulfilled, and the bishop died on the 17th of March, 1714–15.

Making no pretension to a biography of Mead, we shall merely refer to his contributions to medical science and practice. On the application of Mr. Secretary Craggs, in 1719, Mead published a discourse on the Plague, and the most effectual method to prevent the spreading of this disease, which had proved so fatal that year at Marseilles. This brochure, as it could now be called, went though no less than seven editions in a twelvemonth, (1720.) It was distinguished by an erudition which one would not expect to meet in a volume or pamphlet of the present day. He strenuously advocated a system of quarantine, which was adopted by the government. Referring to the fierce opposition which he encountered for this recommendation, he makes use of the following language, which must, just now, be considered apposite to the discussions going on in the United States on the subject, especially in reference to yellow fever: "But suppose for a moment," said he to a friend, "that the laws of quarantine were useless, and that the fears entertained of the nature of the plague were without foundation, how can the commerce of this country be benefited by the abolition of these regulations here, unless the rest of civilized Europe adopt the same measure, and agree as a sort of general congress, to remove all restraints from their trade with the Levant?"

Two or three years after this the attention of the master of the gold-headed cane was called to another question of still greater importance than the one just mentioned. This was no less than the mitigation of that loathsome disease, the small-pox. Lady Mary Wortley Montague, whose husband had been English ambassador at Constantinople, had witnessed the practice of inoculating for the small-pox in the East, and determined to have the operation performed on her own children. This was accordingly done on her son, before leaving the Turkish capital, and on her daughter, after her return home, in 1722, with entire success. The profession was, however, slow to adopt the practice, and further trials, in the interests of royalty, were made on condemned felons. Six of these were inoculated, and five of them took the disease in a benignant form; the sixth was not affected. They were pardoned, as a condition for their consenting to the experiment. At Dr. Mead's suggestion, the Chinese method was practiced on a young girl, eighteen years of age, by introducing a tent moistened with matter taken from the pustules and then introduced into her nostrils. The girl in this case, as in that of the five others, perfectly recovered, after having contracted the disease. Encouraged by these results, the practice of inoculation made a bound from the prison to the palace, and one of the children of the Princess of Wales was inoculated with success. Dr. Mead expresses his surprise that the contagious nature of small-pox, which is so obvious, should not have been noticed by every writer on the subject; and yet Sydenham, discerning as he was, does not take the slightest notice of it. To Mead's treatise on small-pox and measles, which appeared twenty years later, its author added a translation of Rhaze's commentary on the disease, "a copy of which he had obtained from Leyden, through the assistance of his friend and fellow-student Boerhaave, with whom my master kept up a constant correspondence."

Notice is taken of the method first used by Mead of exerting pressure on the abdomen, after the operation of tapping, in order to prevent the fainting and even death itself which, without this precaution, have sometimes ensued. Gold-headed Cane *loquitur:* "I was present when this method was first tried in the hospital, and afterwards frequently saw it used in the case of Dame Mary Page, wife of Sir Gregory Page, Bart., who was afflicted with this disease, and died March 4th, 1728, in the fifty-sixth year of her age. In sixty-seven months she was tapped sixty-six times, and had two hundred and forty gallons of water taken away, without ever once fearing the operation."

Reverting to some particulars in Mead's life of a personal nature, we find that Garth, author of the poem of

"The Dispensary," Arbuthnot, eminent as a physician, a scholar, and a wit, and Freind, author of the "History of Medicine," were among his chief associates. These names are the more noticeable, for they were those of zealous Tories, whereas the holder of the gold-headed cane was a staunch Whig. This fact gives the more point to the warm regard manifested by Mead for Freind, on the occasion of the latter, who was a member of Parliament, and had been complicated in a conspiracy to restore the Stuarts to the throne, being committed to the Tower. Freind's practice fell chiefly into the hands of Mead; and to him was the captive indebted for his liberation from prison. When Sir Robert Walpole, the minister of the day, sent to consult Mead on account of some indisposition, the physician availed himself of the occasion to plead the cause of his friend. He urged the patriotic feelings and loyalty of Freind, his public services, his meriting well of science, his being "besides a man of excellent parts, a thorough scholar and one whom all acknowledged to be very able in his profession,"—and, finally, wound up by an *argumentum ad hominem*, in refusing to prescribe for the minister unless the prisoner was set at liberty. The appeal was successful, and Freind was admitted to bail; his sureties being, Dr. Mead, Dr. Hulse, Dr. Levet, and Dr. Hale. We let the cane tell the following incident, which has generally been regarded as historical, although we know that some have doubts of its having really occurred:—

"The evening after this event there was a numerous assembly at our house in Great Ormond Street, attracted by the hope of meeting Freind, and congratulating him on his liberation from the Tower. He came, and every one was delighted to see him once more at large. Besides the number of acquaintances and friends who were there, when it is observed that no foreigners of any learning, taste, or even curiosity, ever arrived in England without being introduced to my master, (as it would have been a reproach to have returned without seeing a scholar and physician who was in correspondence with all the literati of Europe,) it may easily be imagined that on so remarkable an occasion our *conversazione* was a crowded one. When the party broke up, and Freind and Arbuthnot were about to take their leave together, as they lived in the same part of the town—the former in Albemarle Street, and the latter in Cork Street, Burlington Gardens—Dr. Mead begged Freind to step with him for a moment into his own private study, which was a small room adjoining the library. There he presented him with a sum of five thousand guineas, which he had received from Freind's patients, whom he had visited during his imprisonment. On returning to the great room he wished them both good night, and jocosely said to Arbuthnot, (who happened to hold the office of Censor of the College this year,) 'Now I commit our friend here to your magisterial care and guidance; see that he does not again get into trouble, and on the least appearance of irregularity report him to the President, Sir Hans Sloane. I look to you, Arbuthnot, to preserve harmony among us.' "*

A good idea may be formed of the riches in literature and art that abounded in Dr. Mead's house, and at the same time, of the munificent style in which he received his company, by the fol-

* Arbuthnot was a dilettante in music, and occasionally composed sacred pieces.

lowing talk of our imaginary guide. We wish that the description would serve as an incentive for those of our contemporaries who have the means, if there be such in our great cities, to imitate him in this particular, even though they may fall short of him in learning.

"These meetings, of which Dr. Mead was very fond, took place at stated periods, and the visitors assembled in the library, a spacious room about sixty feet long, of the richness of which an idea may be formed by referring to the catalogue of the sale of its contents, which took place after his death. The books, amounting to ten thousand volumes, were sold in twenty-eight days. The sale of prints and drawings occupied fourteen evenings, and the coins and medals were disposed of in eight days. But at the time of which I speak, all these literary treasures were collected under one roof; and the assemblage of marble statues, of Greek philosophers, Roman emperors, bronzes, gems, intaglios, Etruscan vases, and other rare specimens of antiquity, was most choice and valuable. Ranged along one side of the room stood the busts of the great English poets—Shakspeare, Milton, and Pope: they were of the size of life, of white marble, and by the hand of Scheemakers. The corner in which I was usually placed was between the statue of Hygeia* and a cabinet of iron which once belonged to Queen Elizabeth. This cabinet was full of valuable coins, among which was a medal of the Protector, (Cromwell,) which Mead frequently exhibited as a curiosity to his visitors; it had Oliver's head in profile, with this legend: 'The Lord of Hosts, the word at Dunbar, Sept. 1650;' on the reverse, the parliament sitting.

"Placed in this favorite spot, I often heard," continues the gold-headed narrator, "very interesting discourse. On one occasion, particularly, the conversation turned on the condition and rank of physicians in society—a topic elicited by the attack which had been lately made by Dr. Conyers Middleton on the dignity of medicine, in a dissertation written by him concerning the state of physic in old Rome. Dr. Mead began by asking, 'What class of men have deserved better of the public than physicians? How much, for instance, does not this country owe to Linacre, the founder of our College?'" The sketch drawn of this eminent man, and of his successor, Dr. Caius might well find a place in our table-talk. So also the remarks on Garth, by whose exertions the remains of Dryden had a suitable interment. One of the persons present at this conversation, and who was leaning on the arm of Arbuthnot, is thus described: "He was protuberant before and behind, and used humorously to compare himself to a spider; and was so feeble that he could not, as I have heard, dress or undress himself, and was always wrapped up in fur and flannel, besides wearing a bodice of stiff canvas. In this description every one will recognize the form of Pope. He took no part in

* At the sale of Mead's library and works of art, this statue, three feet and a half high, was bought for £56 sterling, ($250,) by Dr. Anthony Askew. On the same occasion a magnificent statue of Antinous, of white marble and of the size of nature, was purchased by the Marquis of Rockingham for 241 pounds sterling, ($1205.) The celebrated bronze head of Homer was sold for 136 pounds ($680) to Lord Exeter.

the conversation, but his fine, sharp, piercing eye, directed as it was alternately to the different speakers, indicated that he felt no common concern in the subject."

The inspection of a bust of Harvey, which Mead had recently caused to be executed from an original picture in his possession, gave rise to some reminiscences introduced by Freind, of the discoverer of the circulation of the blood. Dr. George Ent gives a pleasing account of a visit which he paid to his friend Harvey, who was then in his seventy-first year, living in retirement at Richmond. In a garden belonging to the College of Physicians, Harvey built at his own expense an elegantly-furnished Convocation Room, and a Museum filled with choice books and surgical instruments. On the second day of February, 1653, he invited the members to a splendid entertainment, and on that occasion presented the College with a deed of gift of the building he had erected in their garden. Worthy of mention here, is a little episode in the narrative of the olden time. In March, 1823, the Earl of Winchelsea presented to the College some anatomical preparations which belonged to his ancestor, Dr. Harvey. These consist of six tables or boards upon which are spread the different nerves and blood-vessels carefully dissected out of the body—in one of them the semi-lunar valves of the aorta are distinctly to be seen. These preparations were most probably by Harvey himself, and he may have learned the art of making them in Italy, for he studied in Padua, in 1602. The original manuscripts of a course of lectures on anatomy and surgery, which he gave before the College of Physicians in 1616, are preserved in the British Museum.

It was my lot, says the gold-headed cane, often to be in company with the eminent surgeon, Cheselden, for the public seemed universally to have adopted the sentiment of the popular poet of the day:—

> "I'll try what Mead and Cheselden advise."
>
> POPE.

The particular case in which these two eminences were called upon for joint professional action, with that of Mr. Conduitt, surgeon in ordinary, was of Sir Isaac Newton, who was said to be suffering from stone in the bladder: "No hopes were given of his recovery; and yet to look upon the great philosopher, though now in his eighty-fifth year, he had the bloom and color of a young man, had never worn spectacles, nor lost more than one tooth during his whole life. Besides being blessed with a very happy and vigorous constitution, he had been very temperate in his diet, though we did not learn that he had ever observed any regimen. He was of middle stature, and at this time plump in his person; had a very lively and piercing eye, a comely and gracious aspect, and a fine head of hair, as white as silver, without any baldness, and when his peruke was off he had truly a most venerable appearance."

On the accession of George the Second, Dr. Mead was made one of the royal physicians, and was for many years engaged in the constant hurry of an extensive and successful practice. Few princes, it was acknowledged, have shown themselves equally generous and liberal in promoting science and encouraging learning. He threw open his gallery in the morning for the benefit of students in painting and sculpture; and was in the habit of even lending the best of his pictures to artists to copy. If any literary work was going on he contributed all in his power to its perfection. Guy, the wealthy citizen, was persuaded by Dr. Mead to lay out his immense fortune in building that hospital in the Borough, which bears his name. With respect to science, no discovery was made in which he did not take a lively interest.

The hospitality of Mead was unbounded, and consequently his housekeeping expenses were very great; for, not content with the reception of his own friends and acquaintances, he kept also a very handsome second table, to which persons of inferior quality were invited. The consequence was, that notwithstanding the considerable gains derived from his profession, (for several years he made between 5000 and 6000, and during one year, 7000 pounds sterling,*) he did not die so rich as might have been expected. The total amount left at his death, including the receipts of the sale of his library, pictures, statues, etc., (which were between 15,000 and 16,000 pounds,) was about 50,000 pounds, ($200,000;) but this sum was materially diminished by the payment of his debts. It is somewhat remarkable that many of his books sold for much more than they cost him. His pictures, also, were chosen with so much judgment, that they produced upwards of $17,000; or about $3000 to $3500 more than he gave for them.

With respect to his manner of living when not engaged at home, he generally spent his evenings at Batson's Coffee-house; and in the forenoons, apothecaries used to come to him at Tom's, near Covent Garden, with written or verbal reports of cases, for which he prescribed without seeing the patient, and took half-guinea (about $2.50) fees.

Mead's last work was entitled "Medical Precepts and Cautions," (*Moneta Medica*,)† in which he freely communicates all the discoveries that the long practice and experience of nearly half a century had opened to him concerning diseases and their cures; and concludes with many salutary directions for preserving the body and mind perfect and entire to a good old age. This he attained himself, and preserved, till within three years of his death, his intellectual powers in a state of perfection. Then he became very corpulent, and his faculties were visibly impaired. Dr. Mead died on the 16th of February, 1754, in his eighty-first year. He was twice married: by his first wife he had ten children, of whom three survived him; his daughters were married to Dr. Wilmot and Dr. Nicholls, and a son, Richard, was heir to his father's and uncle's fortunes. By his second wife he had no issue.

After the death of Dr. Mead, it was said of him, that, of all the physicians who had ever flourished, he gained the most, spent the most, and enjoyed the highest fame during his lifetime, not only in his own, but in foreign countries.

---

FLORENCE NIGHTINGALE.—Florence Nightingale needs but few prefatory remarks. Reared in luxury, and fitted both by natural and acquired endow-

---

* In our currency their amounts would be respectively, 25,000, 30,000, and 35,000 dollars.

† It was preceded by a treatise *De Morbis Biblicis*, or, as called in the English translation executed under the eye of the author, "*Medica Sacra*, or a Commentary on the most remarkable diseases mentioned in the Holy Scriptures."

